# Trends in the Prevalence of Dementia in Japan

**DOI:** 10.1155/2012/956354

**Published:** 2012-10-03

**Authors:** Hiroko H. Dodge, Teresa J. Buracchio, Gwenith G. Fisher, Yutaka Kiyohara, Kenichi Meguro, Yumihiro Tanizaki, Jeffrey A. Kaye

**Affiliations:** ^1^Department of Neurology, Oregon Health & Science University, CR131, 3181 SW Sam Jackson Park Road, Portland, OR 97239-3098, USA; ^2^Department of Epidemiology, University of Pittsburgh Graduate School of Public Health, Pittsburgh, PA 15213, USA; ^3^Department of Neurology, University of Michigan, Ann Arbor, MI 48190, USA; ^4^Institute for Social Research, University of Michigan, Ann Arbor, MI 48106, USA; ^5^Department of Environmental Medicine, Graduate School of Medical Sciences, Kyushu University, Fukuoka, Japan; ^6^Department of Geriatric Behavioral Neurology, Tohoku University Graduate School of Medicine, Sendai, Japan; ^7^Department of Neurology, Portland Veterans Affairs Medical Center, Portland, OR, USA

## Abstract

There is a paucity of data regarding trends in dementia and its subtype prevalence in Japan. Our aims in the current paper are to: (1) summarize epidemiological studies of dementia in Japan including relevant details of study protocol and diagnostic criteria, (2) compare the age-specific prevalence of all-cause dementia among studies, and (3) assess the trends in Alzheimer's disease (AD) versus vascular dementia (VaD) over time. We reviewed diagnostic criteria, all-cause dementia prevalence, and the AD/VaD ratio from 8 large population studies of dementia in Japan. Compared with the Okinawa 1992 study, studies conducted in 1994, 1998, 2005, and 2008 had a higher prevalence of all-cause dementia using Poisson regression models, after controlling for age and sex. In contrast to the US and some European countries, all-cause dementia prevalence is increasing in Japan. The prevalence of AD as opposed to VaD seems to be increasing over time, but large variability in diagnostic criteria, possible regional variability, and differences in prevalence of subtypes of dementia between men and women make it difficult to draw a conclusion about this trend at the national level. Further studies, for example, comparing the population attributable risk of vascular diseases to the prevalence and incidence of dementia could help to clarify the regional variations in etiological subtypes.

## 1. Introduction

It has been reported that the prevalence and incidence of dementia in the United States have been either stable or even declining over the last 2 decades of the 1990s [1]. An important question is what the dementia trends are in other countries. This question is particularly relevant to the case of Japan which is an economically advanced country like the US, but is believed to have a different level of vascular disease risk [2]. Studies conducted in the late 1980s and early 1990s reported that vascular dementia (VaD) was more prevalent than Alzheimer's disease (AD) in Japan, compared with the US and other western countries where AD is more prevalent than VaD [3]. In studies among Japanese Americans conducted in the early 1990s [4, 5], the prevalence ratio of AD to VaD was higher than that reported among Japanese in Japan and more closely resembled that found in the Caucasian population. This suggests that there might have been environmental factors that changed the risks of developing subtypes of dementia after Japanese immigrated to the US. On the other hand, more recent studies conducted in the late 1990s suggest that the cross-national differences found in the past may have been due to differences in the diagnostic methods used [6, 7]. Standardization of diagnosis is one of the challenges of cross-national comparisons of dementia prevalence. There is an ongoing debate as to whether: (1) the higher proportion of VaD found in the past studies in Japan could be due to differences in diagnostic criteria used in Japan and the US, (2) the similar AD/VaD ratio with that of the US found in recent Japanese studies could be due to decreased cerebrovascular disease incidence over the past decades in Japan, or (3) there is no systematic time trend in AD/VaD ratio in Japan and the observed variation is due to regional differences within Japan. Despite a growing interest in the influence of vascular disease and its risk factors on Alzheimer's disease (AD) [8–12], there is a paucity of data regarding dementia prevalence trends in Japan. Our aims in the current study are to: (1) summarize epidemiological studies of dementia in Japan including relevant details of study protocols and diagnostic criteria, (2) compare age-specific prevalence of all-cause dementia among studies, and (3) assess the trends in AD versus VaD over time. Previous studies which concluded an increase in AD/VaD over time in Japan [13–15] did not examine the diagnostic criteria used, nor did they examine age-specific AD/VaD ratios.

## 2. Methods

### 2.1. Study Design and Sample

 We selected dementia prevalence studies in Japan that were designed to be representative of specific communities or prefectures with at least 500 study participants aged 65 and older, and whose age-specific prevalence (for either 5- or 10-year age intervals) for AD and VaD was published between 1990 and 2009 in international journals, using MEDLINE with the search words “Japan,” “dementia,” and “prevalence.” We used the former criterion (*n* ≥ 500) in order to have a large enough sample size to allow meaningful between-cohort comparisons of dementia prevalence. Eight studies met these criteria: the Hisayama study [16, 17] conducted at four time points, the Okinawa study [18], the Radiation Effect Research Foundation Adult Health Survey (RERF-AHS, a.k.a Hiroshima study) [7], the Tajiri Project [6], and the Ama-cho study [14]. Brief descriptions of each study cohort follow (See also Table 1 for further summary).

#### 2.1.1. Hisayama Study

 Hisayama is a rural community adjacent to the city of Fukuoka, a major city of Kyushu Island. An epidemiological study of stroke has been carried out prospectively there since 1961. Cross-sectional dementia prevalence was estimated 4 times, in 1985, 1992, 1998, and 2005 [16, 17]. 

#### 2.1.2. Okinawa Study

 Okinawa is the southernmost island in Japan. Random sampling was conducted to recruit study participants in the selected sites between 1991 and 1992 [18]. 

#### 2.1.3. Radiation Effect Research Foundation Adult Health Survey (RERF-AHS) (Hiroshima Study)

In 1958, the Atomic Bomb Casualty Commission began the Adult Health Study (AHS) to survey the occurrence of illnesses and changes in physiological and biochemical function resulting from exposure to atomicbomb radiation. The original AHS cohort consisted of atomicbomb survivors and their controls, selected from residents in Hiroshima and Nagasaki. Between 1992 and 1996, those aged 60 and older who were residents of Hiroshima and members of the AHS were examined [7].

#### 2.1.4. Tajiri Project

The Tajiri project is a community-based study started in 1988 for the prevention of stroke, dementia, and bed-confinement in Tajiri, an agricultural area in northern Japan. In 1998, all residents 65 years and older were targeted for the dementia prevalence study [6, 20]. 

#### 2.1.5. Ama-cho Study

This study was conducted in the municipality of Ama-cho, a rural island town in the northwestern part of Japan. All residents as of the prevalence day of March 1, 2008 were requested to participate in the dementia prevalence study [14]. 

### 2.2. Statistical Analysis

 Differences in overall dementia prevalence among 8 studies were examined using Poisson regression models based on the number of dementia cases (weighted numbers of cases for Okinawa studies) and the number of participants by 10 year age groups (except the youngest age group where 5-year ages were used: 65–69, 70–79, 80–89, and 90+) to provide large enough sample sizes in each group for meaningful statistical comparisons, yet controlling for changing age composition over time. The Okinawa 1992 study sample was used as a reference group because it was the largest and was conducted at the mid-point of assessment years among the 8 studies. 

## 3. Results

### 3.1. Diagnostic Criteria

Table 1 gives a brief description of each study and the criteria used to define dementia and dementia subtypes. All assessments, except in the Tajiri study, were based on multistage assessments, during which all participants were screened in Phase 1 (screening phase), and selected participants and controls from Phase 1 received final diagnoses from physicians in Phase 2 (clinical assessment phase). The Tajiri study determined dementia diagnoses for all participants. The dementia diagnostic criteria used in Japanese studies were either DSM-III [21], DSM-III-R [22], or DSM-IV [23]. The diagnostic criteria used to define subtypes of dementia varied among the studies in Japan. The following criteria were used to define VaD: Hachinski ischemic scores [24], NINDS-AIREN [25], DSM-IV [23], and the Alzheimer's Disease Diagnostic and Treatment Centers (ADDTC) [26] criteria. For AD, DSM-III-R [22], DSM-IV [23], and NINCDS-ADRDA [27] were used. 

### 3.2. Comparisons of Prevalence of Dementia among Studies

The overall prevalence of dementia among those aged 65 and older ranged from 5.6% (Hisayama 1992) to 11.3% (Ama-cho 2008) (Table 1). Poisson regression models for dementia prevalence showed that compared with the Okinawa 1992 study, Hiroshima 1996 (*P* = 0.0002), Tajiri 1998 (*P* < 0.0001), Hisayama 2005 (*P* < 0.0001), and Ama-cho 2008 (*P* = 0.007) had a higher prevalence of all-cause dementia, controlling for sex and age groups (Table 2). There were no difference between the Okinawa 1992 study and Hisayama studies conducted in 1985, 1992, and 1998. No difference was found between men and women.

 As a post hoc analysis, we also ran Poisson regression models using only the Hisayama studies (4 time points). The results showed that compared with the all-cause dementia prevalence in 1985, that of 2005 was higher (coefficient: 0.32, 95% CI: 0.01–0.64, *P* = 0.04) controlling for sex and age groups (not shown in Table 1).

### 3.3. AD/VaD Ratios

 AD/VaD ratios among those aged 65 and older are listed in the last column of Table 1. The Okinawa (1992), Hiroshima (1996), Tajiri (1998), and Ama-cho (2008) studies used the same criteria for diagnoses of AD (NINCDS-ADRDA) and the AD/VaD ratio among those aged 65 and older was 1.85, 1.85, 3.33, and 4.12 for the above studies, respectively. The Hisayama studies which conducted 4 cross-sectional studies using the same diagnostic criteria for subtypes of dementia also showed the increasing trend in the ratio of AD/VaD, ranging from 0.52 in 1985 to 1.96 in 1998, and 1.92 in 2005. The Tajiri project, which conducted an MRI substudy to identify dementia etiologies, showed that the proportion of AD among total dementia cases differed largely depending on the criteria used: 62.5% (AD/VaD = 3.3) using NINDS-AIREN criteria, and 40.6% (AD/VaD = 1.0) or 56.2% (AD/VaD = 2.3) depending on assessors using DSM-IV criteria. 

 Even though we selected studies with relatively large sample sizes (*n* ≥ 500), the number of cases was too small to conduct meaningful comparisons of age-specific AD/VaD ratios for the age group 65–70 (<5 for each case). Therefore, we list age-specific prevalence of dementia and AD/VaD ratios for the age groups 70–79, 80–89, and 90 and older in Table 3. The youngest age group examined here (age 70–79) had a prevalence of VaD that was consistently higher than that of AD in Japan (i.e., AD/VaD < 1) except in more recent studies conducted in 2005 (Hisayama) and 2008 (Ama-cho). Except for these two recent studies (Hisayama 2005 and Ama-cho 2008), as age increased, the proportion of AD among the total dementia cases increased.

## 4. Discussion 

### 4.1. Overall Dementia

Eight major prevalence studies conducted in Japan were reviewed in an attempt to identify trends in prevalence of all-cause dementia and subtypes of dementia, paying careful attention to diagnostic protocols. We found that compared with the Okinawa 1992 study, studies conducted in later years (1994 (Hiroshima), 1998 (Tajiri), 2005 (Hisayama), and 2008 (Ama-cho)) had a higher prevalence of all-cause dementia, after controlling for age groups and sex. Within 4 studies conducted in Hisayama (1985, 1992, 1998, and 2005), we also found that the prevalence in 2005 was higher than that in 1985, after controlling for age groups and sex. Thus, the dementia prevalence seems to be increasing in Japan, in contrast to the US where decreasing or stable prevalence of all-cause dementia has been reported [1]. 

 A number of reasons may explain the observed trends. Two diseases that could have high impact on the prevalence of dementia at national levels are cerebrovascular disease and type 2 diabetes, as shown by their relatively high population attributable risk % (PAR%) of dementia in the United States [12, 28]. According to the National Nutrition Survey in Japan, those with hemoglobin A1C values ≥ 6.0% (possible type 2 diabetes) were estimated to be 22.8%, 37.4%, and 40.9% among men aged 70 and older in year 1997, 2002 and 2007, respectively, and the comparative figures among women were 27.2%, 28.2%, and 34.6% [29]. On the other hand, the decline in stroke incidence reached plateaus around the late 1990s after a continuous sharp decline beginning in 1960 [30], thus further declines in dementia due to stroke would not be expected after the 1990s. However, it is possible that the prevalence of small vessel cerebrovascular disease with resultant microinfarcts that would not be accounted for in these vascular disease statistics could play a latent or underappreciated role in causing or contributing to dementia. The increase in type 2 diabetes, the metabolic syndrome and its associated vascular complications (risk factors for AD [31]), with a plateau in declining trends in major stroke incidence, could have lead to an increase in dementia prevalence in recent years in Japan. It is also possible that increasing public awareness of dementia in recent years is resulting in enhanced recognition of functional and cognitive declines that might previously have been dismissed as “normal aging.” Longer survival of those who suffered from stroke/TIA due to advanced medical treatment could also increase the prevalence of dementia to some extent. Unfortunately, autopsy confirmation in large proportions of the participants in these epidemiologic studies is not available, thus limiting conclusions as to more specific underlying etiologies.

 Our finding could be also partly due to regional variability in prevalence of dementia. We found that Okinawa had a lower overall prevalence of dementia compared with other cohorts except Hisayama studies in 1985, 1992, and 1998. These two regions (Okinawa and Hisayama) have had lower incidence of cerebrovascular diseases in comparison with other regions studied here. For example, the rate of cerebrovascular mortality, as a proxy of cerebrovascular disease incidence, declined sharply between 1975 and 2000 by over 65% in all regions and declined further between 2000 and 2005 by over 10% in all regions (Table 4), with the above two regions constantly having lower rates than other regions. Historically, salt consumption has been high in the northern part of Japan because vegetables and marine products are cured with salt to preserve them during the longer winter months and consumed with processed white rice. This dietary pattern is believed to be one of the reasons of the higher prevalence of hypertension and large vessel cerebrovascular disease in the northern prefectures in Japan [32]. The low dementia prevalence in Okinawa and Hisayama (except 2008), which is located in the southern part of Japan, could not only be due to a lower rate of VaD resulting from lower cerebrovascular disease incidence, but also due to lower AD prevalence resulting from reduced vascular injuries [8, 12, 33, 34].

### 4.2. AD versus VaD

 Vascular dementia (VaD) was believed to be more prevalent than Alzheimer's disease (AD) in Japan in the 1980s, in contrast to the US or other western countries, but studies conducted in the late 1990s and after showed patterns that were more similar to the US [13, 15, 35]. This “westernization” of the dementia prevalence pattern could be partly due to the declining stoke incidence observed during the 1980s as described above. However, it could also be due to changes in diagnostic criteria used in Japan. For example, in the Tajiri study, patients received a diagnosis of “possible AD with CVD” by means of the NINDS-AIREN criteria, provided that the vascular effect on dementia was considered to be too ambiguous to diagnose as VaD [6], which lead to relatively high proportion of AD out of all dementia cases (over 62%). The study also demonstrated the difficulty of obtaining consensus on the definition of VaD, even for experienced neurologists using the same criteria; two neurologists blinded to each other's diagnosis did not agree when diagnosing VaD versus AD under DSM-IV criteria: the proportion of VaD out of total dementia cases ranged from 40.6% to 56.2%, depending on assessors. We expect that lack of imaging data may result in underdiagnosis of subcortical vascular brain injury (e.g., white matter hyperintensity, and silent brain infarcts, etc.) and thus lead to underestimation of VaD. However, more recent studies including the Tajiri, Hisayama (1998; 2005), Hiroshima and Ama-cho studies used imaging data, and these tend to show higher AD/VaD ratios (i.e., not higher VaD prevalence) compared with earlier studies. Therefore, it is likely that changes in diagnostic criteria at least partly explain the higher proportion of diagnosed AD in recent years. 

 In all studies except more recent studies conducted after 2000, as age increased, the proportion of AD among the total dementia cases increased: the youngest age group examined (age 70–79) had a prevalence of VaD that was consistently higher than that of AD in Japan (i.e., AD/VaD < 1). On the other hand, among the older age groups, AD had been more prevalent. In fact, among the oldest age group (age ≥ 90), AD/VaD ratios in Japan were not necessarily lower than the US figures even among studies conducted in the early 1990s, ranging from 2.5 (Hisayama 1985) to 6.0 (Hisayama 1992). In the Aging, Demographics, and Memory Study (ADAMS) [36, 37], which is the first study in the US to calculate a nationally representative dementia prevalence, the age-specific AD/VaD ratios were found to be 2.36, 4.43, and 4.79 for age group 71–79, 80–89, 90 and older, respectively, using DSM-III-R [22] and DSM-IV [23] criteria for dementia and NINCDS-ADRDA criteria for AD, that is, the age-associated increase in AD prevalence seems to be more evident in Japan than in the US. This could be partly due to the fact that the gender gap in life expectancy is larger in Japan compared with US and the proportion of women increases more steeply as age increases in Japan. For example, in 1990, the life expectancy at age 65 was 18.9 years for women and 15.1 years for men in the US, that is, a gender gap of 3.8 years, while the comparative figure in Japan was 20.0 years and 16.2 years, with the gender gap of 4.8 years. Similarly, in 2008, the life expectancy at age 65 was 19.8 years for women and 17.1 years for men in the US, with the gender gap of 2.7 years, while the comparative figure in Japan was 23.6 years and 18.6 years, with the gender gap of 5.0 years. Cerebrovascular disease is more common among men than women, and we expect a higher proportion of VaD among men than women. A steeper age-associated increase in AD prevalence found in Japan, therefore, could be due to a higher proportion of women among the older old age groups in Japan. Comparisons of age-specific AD/VaD ratios between men and women could clarify this issue, but we were unable to do so due to the small sample size once we stratify prevalence by sex and age groups, especially among those aged 90 and older. However, at least among the younger two age groups (ages 70–79; 80–89), we see an increasing trend in the proportion of AD cases as age group goes up in both men and women (data not shown). These results also suggest that it is important to consider the age/sex structure of samples when we compare the AD/VaD ratio among different cohorts. The comparison of aggregated ratios could be misleading. 

### 4.3. Incidence of Dementia

 Although incidence of dementia and its subtype would give a more accurate picture regarding the trend, there is a paucity of dementia incidence studies in Japan. To our knowledge, only three incidence studies have been reported thus far: (1) the Hisayama cohort, following their 1985 cohort for 7 years [38]; (2) the Tajiri project, following a sub-sample of their 1998 cohort [39] for up to 7 years; (3) the Hiroshima study (Radiation Effects Research Foundation adult health study), following their 1992–1996 prevalence cohort until the year 2003 [40]. The incidence of all-cause dementia was 19.3 per 1000 person-years for men and 20.9 for women in the Hisayama study, 33.9 for men and 44.0 for women in the Tajiri study, and 12.0 for men and 16.6 for women in the Hiroshima study. The Hisayama and Hisorhisma studies reported significant gender differences in subtypes of dementia incidence: Hisayama reported that the incidence of VaD increased with age and was consistently higher than that of AD for men, while the incidence of AD was higher than that of VaD for women age 75 years or older. The incidence of AD markedly increased after the age of 80 in either sex, but overall, VaD was more common in the Hisayama study. The Hiroshima study also reported that probable AD showed the most remarkable increase with age, and probable VaD was significantly lower among women. Overall, AD was more common in this study, possibly because the Hiroshima study was conducted later than the Hisayama study, after a further decline in stroke incidence had occurred. In all 3 incidence studies, NINCDS-ADRDA criteria were used for AD and NINDS-AIREN for VaD. In Japan, AD incidence and prevalence tend to increase with age more than VaD and older old women predominantly have AD rather than VaD. Therefore, when aggregated, the overall prevalence of AD may become higher as more women survive in the oldest old age group. As we mentioned earlier, it is important to consider the age/sex structure of samples when examining trends in the AD/VaD ratio. 

### 4.4. Conclusion

 In conclusion, our systematic review shows that (1) there is an increasing trend in overall dementia in Japan. Although we cannot confirm definitively from the current study, the possible explanation of the increase could be a shift in health conditions among the elderly in Japan including the increase in diabetes mellitus in more recent years despite the plateau in decline in stroke incidence during the late 1990s; (2) the similar AD/VaD ratio found in recent studies in Japan with that of the US could be due to a combination of at least 3 factors: (a) shifting diagnostic criteria (more in line with US consensus diagnosis), (b) possible shifting in health conditions among the elderly in Japan (decline in stroke incidence, but increase in metabolic disease e.g., type 2 diabetes, hyperlipidemia, and atherosclerosis), (c) an increase in the proportion of the oldest old who had an historically higher prevalence of AD (as opposed to VaD) in Japan, and (d) regional variations (i.e., a north-to-south gradient in VaD) possibly due to large difference in dietary patterns. 

 The study limitations include relatively small sample sizes, especially for the oldest old group. The Okinawa study took into account the potential dementia cases among those who were screened at Phase 1 (screening phase), but did not participate in Phase 2 (clinical assessment phase), using weights generated from Phase 1. However, as with most other epidemiological studies of dementia, there was no way of precisely estimating the frequency of dementia among those who did not participate in the screening phase. Four Japanese prevalence studies [15, 41–43] were not included in this study because the age-specific prevalence of AD and VaD were not provided in the published articles. Although it would be interesting to deconstruct the changes in all-cause-dementia prevalence and AD/VaD ratios into the potential explanatory factors, different criteria used to define subtypes of dementia would not allow this type of quantitative assessment. One of the strengths of our study is that we could ascertain the detailed screening procedures and diagnostic criteria by contacting the investigators of the original studies.

### 4.5. Future Directions

Future studies could aid in monitoring changing prevalence patterns and their causes by including some of the following elements. First, as with Hisayama studies, it would be ideal for studies to examine the prevalence of dementia and its subtypes repeatedly at the same locations using the same criteria. Second, it would be helpful if future epidemiology studies would recruit more participants aged 90 and older (e.g., through aggressive age-stratified sampling protocols) to improve estimates of prevalence in this age group. Third, more comprehensive diagnostic criteria should be used for inter-cohort comparisons: as suggested by Viswanathan and colleagues [8], AD and VaD exist in a continuum of disease. It might be more meaningful if we could apply, for example, more specific clinicopathological criteria for mixed dementia [44–50] than those currently used. Increased use of standardized neuroimaging such as the Alzheimer's Disease Neuroimaging Initiative (ADNI) [51] might aid in the development of more specific and comparable criteria for the diagnosis of VaD. Finally, autopsy confirmation of the underlying potential causes of dementia in epidemiological studies would go a long way to help resolve these important uncertainties in the shifting patterns over time of the dementias.

## Figures and Tables

**Table 1:** Description of each study in Japan.

Study name/site (study years)	Sampling frame	Assessment protocol	Prevalence of all-cause dementia among those aged 65 and older:
			(1) Prevalence in percentage (95% CI)
			(2) Number of cases (all subtypes combined/AD/VaD)
			(3) AD percentage out of all dementia
			(4) AD/VaD ratio
Hisayama (1985, 1992, 1998, and 2005)	Total residents aged ≥ 65 in the area: Year 1985: *N* = 938 Year 1992: *N* = 1231 Year 1998: *N* = 1442 Year 2005: *N* = 1711	Phase 1 Suspected dementia cases selected using Hasegawa dementia scale, neuropsychological tests, and ADL	Year 1985 (1) 6.7 (5.0–8.3) (2) 59/12/21 (3) 20.3% (4) 0.57
	Study participants at Phase 1 (participation rates) Year 1985: *N* = 887 (94.6%) Year 1992: *N* = 1189 (96.6%) Year 1998: *N* = 1437 (99.7%) Year 2005: *N* = 1566 (91.5%) Sampling weights used to reflect prevalence of population at large? (Yes/No): No	Phase 2 DSM-III-R, Karasawa's criteria (supplementation), and Hachinski's evaluation scale for cerebral ischemia in case vascular effect on dementia were considered to be too ambiguous to diagnose as VaD using the following guidelines: Hachinski's ischemic score <4.0—AD ≥8—VaD 5, 6, 7—mixed/other	Year 1992 (1) 5.6 (4.4–7.1) (2) 68/21/22 (3) 30.8% (4) 0.95 Year 1998 (1) 7.1 (5.7–8.5) (2) 102/49/25 (3) 48.0% (4) 1.96 Year 2005 (1) 12.5 (10.7–14.2) (2) 195/96/51 (3) 49.2% (4) 1.89

Okinawa (1991-1992)	Randomly sampled one city from the urban districts and one town/village from the rural districts from each of 5 regions covering the entire Okinawa prefecture. Randomly selected approximately 17% of the residents from the selected cities and towns/villages in each region.	Phase 1 Pilot study was conducted (*N* = 789) to examine the best MMSE cut-point for screening. MMSE score of 16 had the maximum combination of sensitivity and specificity to identify the demented in the community Selected those with MMSE ≤16 (*N* = 482)	(1) 6.7 (3.6–7.8) (2) 170/80/53 (3) 47.1% (4) 1.51
	Study participants at Phase 1: *N* = 3524 Sampling weights used to reflect prevalence of population at large? (Yes/No): Yes	Phase 2 (*N* = 482) DSM-III-R for dementia, NINCDS-ADRDA for AD, and Hachinski's ischemic score as a guideline for VaD	

Hiroshima (Radiation effect research foundation Adult Health Survey ((RERF-AHS)) (1992–1996)	Residents in Hiroshima among the Original AHA cohort (atomicbomb survivors in Hiroshima and Nagasaki and their controls followed since 1958) evaluated by biennial physical exams between 1992 and 1996. Targeted *N* = 2934	Phase 1 Subjects with CASI*≤ 75 (*N* = 339) and controls with CASI > 75 (*N* = 276) were selected	(1) 8.5 (7.2–9.8) (2) 156/74/40 (3) 47.4% (4) 1.85
	Study participants at Phase 1: *N* = 2222 Sampling weights used to reflect prevalence of population at large? (Yes/No): No	Phase 2 (*N* = 550) DSM-III-R for dementia, NINCDS-ADRDA for AD, ADDTC for ischemic vascular dementia, and DSM-III-R for VaD	

Tajiri Project (1998)	All residents in Tajiri town aged ≥ 65, targeted *N* = 3207	All (*N* = 1654) were evaluated by CDR & DSM-IV (not multistage sampling)	(1) 8.5 (7.2–9.9) Based on subtype analysis with MRI (I) Using NINCDS-ADRDA, NINDS-AIREN (2) 32/20/6 (3) 62.5% (4) 3.33
	Study participants *N* = 1654 Subsample selected for dementia subtype identification study, targeted *N* = 564 Sampling weights used to reflect prevalence of population at large? (Yes/No): No	MRI study: 564 selected randomly from 1654 above, of whom 497 participated in MRI study (dementia subtype identification study). Comparisons of prevalence of VaD using 3 different criteria: (1) DSM-IV for AD and VaD; (2) NINCDS-ADRDA for probable AD, NINDS-AIREN for possible AD with CVD and probable VaD; (3) ADDTC for probable ischemic vascular dementia	(II) Using DSM-IV for AD and VaD (2a) 32/13/13 (2b) 32/18/8 (3a) 40.6% (3b) 56.2% (4a) 1.00 (4b) 2.25 The difference between (a) and (b) above is due to diagnostic differences between assessors

Ama-cho (2008)	All residents in Ama-cho aged ≥ 65, targeted *N* = 943	Phase 1 (*N* = 917) Suspected dementia cases selected through interviews with subjects and their informants, which assessed cognitive changes, psychiatric symptoms, personality changes, problem behaviors, activities of daily living, psychological and medical symptoms, and through assessments of the subjects' medical history offered by the home doctors of the subjects	(1) 11.3 (9.1–13.2) (2) 104/66/16 (3) 63.5% (4) 4.12
	Study participants *N* = 917 (after excluding 23 subjects out of 943 subjects who resided out of town at the time of phase 1 interview) Sampling weights used to reflect prevalence of population at large? (Yes/No): No	Phase 2 (*N* = 120) DSM-IV for dementia, NINCDS-ADRDA for AD, and NINDS-AIREN for VaD	

*CASI: The Cognitive Abilities Screening Instrument [19].

**Table 2:** Factors associated with all-cause dementia prevalence.

	Coefficient (*P* value)	95% confidence interval
Age group		
65–69 (reference)	0	
70–79	1.24 (<0.0001)	1.14–1.34**
80–89	2.62 (<0.0001)	2.52–2.71**
90 and +	3.51 (<0.0001)	3.41–3.62**
Sex		
Women (reference)	0	
Men	−0.01 (0.50)	−0.06–0.03
Study name (year)		
Hisayama 1985	0.16 (0.25)	−0.11–0.43
Hisayama 1992	−0.02 (0.85)	−0.26–0.21
Okinawa 1992 (reference)	0	
Hiroshima 1996	0.30 (0.0002)	0.14–0.46**
Hisayama 1998	0.08 (0.39)	−0.11–0.28
Tajiri 1998	0.37 (<0.0001)	0.19–0.55**
Hisayama 2005	0.49 (<0.0001)	0.35–0.64**
Ama-cho 2008	0.27 (0.007)	0.07–0.47**

Results are based on a Poisson regression model where all study data are included in one model, with the Okinawa study being a reference.

**Significant at *P* < 0.01.

**Table 3:** Prevalence of dementia (%) and its subtype by 10 years age group and by study site.

	Age 70–79		Age 80–89		Age 90 and older	
Study name/site	Prevalence in percentage (95% CI)	AD/VaD ratio	Prevalence in percentage (95% CI)	AD/VaD ratio	Prevalence in percentage (95% CI)	AD/VaD ratio
(Study years)	(Number of subjects screened)	Number of cases: all subtypes combined/AD/VaD	(Number of subjects screened)	Number of cases: all subtypes combined/AD/VaD	(Number of subjects screened)	Number of cases: all subtypes combined/AD/VaD
Hisayama (1985)	3.41 (1.92–5.56)	AD/VaD = 0.13	19.50 (13.65–26.52)	AD/VaD = 0.75	57.14 (28.86–82.34)	AD/VaD = 2.50
	(440)	15/1/8	(159)	31/6/8	(14)	8/5/2
Hisayama (1992)	3.35 (2.00–5.25)	AD/VaD = 0.22	13.39 (9.34–18.37)	AD/VaD = 1.44	46.43 (27.51–66.13)	AD/VaD = 6.00
	(537)	18/2/9	(239)	32/13/9	(28)	13/6/1
Okinawa (1991-1992)	3.67 (2.32–5.02)	AD/VaD = 0.59	14.93 (10.97–18.89)	AD/VaD = 2.10	36.83 (28.08–45.58)	AD/VaD = 3.66
	(1481)*	45/16/27	(619)*	81/42/20	(117)*	37/22/6
Hiroshima (1992–1996)	5.67 (4.09–7.66)	AD/VaD = 0.93	19.04 (14.97–23.87)	AD/VaD = 2.64	41.67 (26.97–61.50)	AD/VaD = 4.66
	(741)	42/13/14	(394)	75/45/17	(60)	25/14/3
Hisayama (1998)	3.99 (2.65–5.76)	AD/VaD = 1.71	16.84 (12.76–21.59)	AD/VaD = 1.92	35.59 (23.55–49.13)	AD/VaD = 3.33
	(676)	27/12/7	(297)	50/25/13	(59)	21/10/3
Tajiri (1998)	5.18 (3.80–6.93)	AD/VaD = N/A	22.33 (17.37–28.26)	AD/VaD = 1.50	51.52 (30.00–82.51)	AD/VaD = N/A
	(908)	7/3/0^#^	(309)	22/12/8^#^	(33)	N/A
Hisayama (2005)	6.86 (5.13–8.94)	AD/VaD = 2.40	27.74 (20.50–29.37)	AD/VaD = 1.84	53.49 (42.41–64.32)	AD/VaD = 1.71
	(729)	50/24/10	(384)	95/46/25	(86)	46/24/14
Ama-cho study (2008)	6.7 (4.12–9.02)	AD/VaD = 1.75	17.8 (14.81–26.41)	AD/VaD = 11.33	41.3 (26.51–61.52)	AD/VaD = 4.50
	(432)	29/14/8	(275)	49/34/3	(58)	24/18/4

AD/VaD: the number of AD cases/the number of VaD cases.

^#^Based on subsample analysis with MRI results.

N/A: sample size is too small to perform the calculation (AD + VaD cases < 5).

*Random sampling was used for screening. *N* reported is not weighted. Prevalence, proportions of subtypes of dementia, and AD/VaD ratio used weighted frequencies to show those of the population at large.

**Table 4:** Cerebrovascular mortality for the selected prefectures in Japan (per 100,000).

	Men			Women		
		Year			Year		
Prefecture* (latitude)	1975	2000	2005	1975	2000	2005

Miyagi (Tajiri Project site) (38°)	363.1	92.6	81.4	243.2	51.9	44.7
Tottori (Ama-cho study site) (35°)	260.3	78.4	65.9	177.1	49.1	37.6
Hiroshima (RERF-AHS study site) (34°)	222.0	68.6	55.4	162.1	39.9	31.9
Fukuoka (Hisayama study site) (33°)	248.6	68.1	55.0	159.0	42.4	30.4
Okinawa (26°)	190.4	63.5	51.9	113.3	30.0	23.1

(Reference: Japan Ministry of Welfare and Labor. Available at: http://www.mhlw.go.jp/toukei/saikin/hw/jinkou/other/00sibou/toukei.html#hyo2 as of February 1, 2012).

*Listed from north to south prefectures in order.

Years are limited to 1975, 2000, and 2005 when the data is publicly available.
